# Diversity of Pea-Associated *F. proliferatum* and *F. verticillioides* Populations Revealed by *FUM1* Sequence Analysis and Fumonisin Biosynthesis

**DOI:** 10.3390/toxins5030488

**Published:** 2013-03-07

**Authors:** Agnieszka Waśkiewicz, Łukasz Stępień, Karolina Wilman, Piotr Kachlicki

**Affiliations:** 1 Department of Chemistry, Poznan University of Life Sciences, Wojska Polskiego 75, Poznań 60-625, Poland; 2 Institute of Plant Genetics, Polish Academy of Sciences, Strzeszyńska 34, Poznań 60-479, Poland; E-Mails: lste@igr.poznan.pl (Ł.S.); kwil@igr.poznan.pl (K.W.); pkac@igr.poznan.pl (P.K.)

**Keywords:** *FUM* cluster, fumonisins, *Fusarium proliferatum*, *Fusarium verticillioides*, pea seeds, phylogenetic analysis

## Abstract

*Fusarium proliferatum* and *F. verticillioides* are considered as minor pathogens of pea (*Pisum sativum* L.). Both species can survive in seed material without visible disease symptoms, but still contaminating it with fumonisins. Two populations of pea-derived *F. proliferatum* and *F. verticillioides* strains were subjected to *FUM1* sequence divergence analysis, forming a distinct group when compared to the collection strains originating from different host species. Furthermore, the mycotoxigenic abilities of those strains were evaluated on the basis of *in planta* and *in vitro* fumonisin biosynthesis. No differences were observed in fumonisin B (FB) levels measured in pea seeds (maximum level reached 1.5 μg g^−1^); however, in rice cultures, the majority of *F. proliferatum* genotypes produced higher amounts of FB_1_–FB_3_ than *F. verticillioides* strains.

## 1. Introduction

Pea (*Pisum sativum* L.) is one of the major legume crops, which are bred mainly for their high content of proteins present in pea seeds [1] and their valuable amino acid composition. Fungal diseases are frequently occurring factors limiting yield and quality of seed used for food and feed. Still, *Fusarium* pathogens are considered to have minor significance [2,3], while *Ascochyta* sp. and *Alternaria* sp. play major roles in this field [4,5,6]. However, *Fusarium proliferatum* and *F. verticillioides* should not be overlooked as both species are able to produce efficiently a group of the most dangerous *Fusarium* mycotoxins—fumonisins [7,8,9,10]. They are a family of polyketide derivatives, structurally related to sphinganine, compounds disrupting sphingolipid metabolism, causing different toxicological effects in humans, animals, as well as plants [7]. The most abundant fumonisin produced in nature is fumonisin B_1_ (FB_1_), a suspected risk factor for esophageal [11] and liver [12] cancers, neural tube defects [13] and cardiovascular problems [14]. Taking into consideration the available toxicological evidence, the International Agency for Research on Cancer classified FB_1_ as probably carcinogenic to humans (class 2B carcinogen) [15].

Numerous studies have confirmed the presence of fumonisins in plant material contaminated with their producers, vast majority was focused on maize [16,17,18,19]. However, rice and sorghum are also often infected with *Fusaria* belonging to the *Gibberella fujikuroi* species complex: *F. fujikuroi*, *F. proliferatum*, *F. verticillioides* and *F. andiyazi* [20,21]. Several reports describing the contamination of crop plants with fumonisin-producing *Fusarium* species included wheat [22,23], garlic [24,25,26,27], asparagus [26,28,29,30,31], pineapple [26,32] and soybean [33]. 

Until now, little information was provided on pea plants serving as hosts for fumonisin producers and a potential serious threat to human health posed by the contamination of seeds. On the contrary, *F. oxysporum*, *F. solani*, *F. avenaceum* and *F. poae* have been considered as major pathogens of this crop [2]. None of these species is capable of synthesizing fumonisins [7,9]. Many recent studies were concentrated on the variability of *F. proliferatum* and *F. verticillioides* populations occurring in the environment, especially in the geographical and ecological context [8,34,35,36,37]. The latest findings suggest that mycotoxin biosynthetic genes represent good targets to design molecular tools for evolutionary and phylogenetic research with the essential genes from the trichothecene, fumonisin and enniatin/beauvericin metabolic pathways being exploited particularly frequently [26,28,38,39,40,41,42]. Genes encoding the zearalenone and bikaverin pathways are also gaining more attention [41,43]. The high level of sequence divergence among mycotoxin biosynthetic genes (especially *TRI* and *FUM* genes) can be applied to distinguish the populations even on a sub-specific (or even host-specific) level [26], being often more valuable phylogenetic markers for the evaluation of the *Fusarium* species diversity. 

The main aim of this study was to analyze the sequence divergences of the translation elongation factor 1 alpha (*tef*-1α) and *FUM1* genes (encoding the essential enzyme of the fumonisin biosynthetic pathway—polyketide synthase) in two populations of *F. proliferatum* and *F. verticillioides* originating from several Polish pea varieties, compared to the collection strains of both species obtained from different host species. Moreover, the abilities of the selected strains to produce fumonisins *in vitro* were evaluated together with the contamination of the pea seed material with those mycotoxins.

## 2. Results

Seeds of twelve cultivars of pea were screened for presence of the pathogenic fungi. Each genotype was grown in four replicates and two distinct localities in Central Poland: Radzików and Wiatrowo. Fungal species were identified morphologically using optical microscope and only samples containing *Fusarium* species were included in the study. Two cultivars appeared to contain fumonisin-producing *Fusaria* in more than one replicate and in both localities: EZOP and TURNIA. Furthermore, several other cultivars also contained the species of interest: EUREKA, SOKOLIK, TARCHALSKA and WIATO. *F. proliferatum* was predominantly occurring on cv. TURNIA and *F. verticillioides* on cv. EZOP, EUREKA and TURNIA (Table 1). Apart from both studied species, *F. poae*, *F. equiseti*, *F. acuminatum*, *F. avenaceum*, *F. graminearum* and *F. sporotrichioides* were identified occasionally in plant tissues (results not shown). 

**Table 1:** Strains of *F. proliferatum* and *F. verticillioiedes* purified from pea seedsof different cultivars grown in 2011 in two localities in Poland (R: Radzików, W: Wiatrowo), as well as collection strains used in the phylogenetic analyses.

**Strain**	**Species**	**Host/cultivar/locality**	**Year**	**Origin**
KF 3758	*F. proliferatum*	*P. sativum/*SOKOLIK/W	2012	Poland
KF 3759	*F. proliferatum*	*P. sativum/*TARCHALSKA/W	2012	Poland
KF 3735	*F. proliferatum*	*P. sativum/*TURNIA/R	2012	Poland
KF 3736	*F. proliferatum*	*P. sativum/*TURNIA/R	2012	Poland
KF 3737	*F. proliferatum*	*P. sativum/*TURNIA/R	2012	Poland
KF 3738	*F. proliferatum*	*P. sativum/*TURNIA/R	2012	Poland
KF 3731	*F. proliferatum*	*P. sativum/*TURNIA/R	2012	Poland
KF 3733	*F. proliferatum*	*P. sativum/*TURNIA/R	2012	Poland
KF 3734	*F. proliferatum*	*P. sativum/*TURNIA/R	2012	Poland
KF 3763	*F. verticillioides*	*P. sativum/*WIATO/W	2012	Poland
KF 3764	*F. verticillioides*	*P. sativum/*WIATO/W	2012	Poland
KF 3765	*F. verticillioides*	*P. sativum/*WIATO/W	2012	Poland
KF 3661	*F. verticillioides*	*P. sativum/*EUREKA/R	2012	Poland
KF 3740	*F. verticillioides*	*P. sativum/*EUREKA/R	2012	Poland
KF 3660	*F. verticillioides*	*P. sativum/*EUREKA/R	2012	Poland
KF 3766	*F. verticillioides*	*P. sativum/*EZOP/W	2012	Poland
KF 3767	*F. verticillioides*	*P. sativum/*EZOP/W	2012	Poland
KF 3768	*F. verticillioides*	*P. sativum/*EZOP/W	2012	Poland
KF 3769	*F. verticillioides*	*P. sativum/*EZOP/W	2012	Poland
KF 3770	*F. verticillioides*	*P. sativum/*EZOP/W	2012	Poland
KF 3771	*F. verticillioides*	*P. sativum/*EZOP/W	2012	Poland
KF 3772	*F. verticillioides*	*P. sativum/*EZOP/W	2012	Poland
KF 3773	*F. verticillioides*	*P. sativum/*EZOP/W	2012	Poland
KF 3774	*F. verticillioides*	*P. sativum/*EZOP/W	2012	Poland
KF 3775	*F. verticillioides*	*P. sativum/*EZOP/W	2012	Poland
KF 3776	*F. verticillioides*	*P. sativum/*EZOP/W	2012	Poland
KF 3778	*F. verticillioides*	*P. sativum/*TARCHALSKA/W	2012	Poland
KF 3760	*F. verticillioides*	*P. sativum/*TURNIA/W	2012	Poland
KF 3761	*F. verticillioides*	*P. sativum/*TURNIA/W	2012	Poland
KF 3781	*F. verticillioides*	*P. sativum/*TURNIA/W	2012	Poland
KF 3782	*F. verticillioides*	*P. sativum/*TURNIA/W	2012	Poland
**Strain**	**Species**	**Collection strains**	**Year**	**Origin**
CBS 513.88	*A. niger*	NT_166526.1		
FOXG_03515	*F. oxysporum*			
FVEG_02381	*F. verticillioides*	*Z. mays*		
KF 497	*F. proliferatum*	*T. aestivum*	1987	Portugal
KF 925	*F. proliferatum*	*Z. mays*	1986	Poland
KF 3441	*F. proliferatum*	*Z. mays*	2006	Poland
KF 3654	*F. proliferatum*	*Z. mays*	2011	Poland
KF 3616	*F. proliferatum*	*A. cepa*	2011	Poland
KF 3385	*F. proliferatum*	*A. comosus*	2009	Vietnam
KF 3548	*F. proliferatum*	*A. comosus*	2011	Ecuador
KF 3550	*F. proliferatum*	*A. comosus*	2011	Ecuador
KF 3355	*F. proliferatum*	*A. officinalis*	2009	Poland
KF 3357	*F. proliferatum*	*A. officinalis*	2009	Poland
KF 3362	*F. proliferatum*	*A. officinalis*	2009	Poland
KF 3360	*F. proliferatum*	*A. officinalis*	2009	Poland
KF 3369	*F. proliferatum*	*A. sativum*	2009	Poland
KF 3372	*F. proliferatum*	*A. sativum*	2009	Poland
KF 3503	*F. proliferatum*	*A. sativum*	2010	Poland
KF 3409	*F. proliferatum*	*Cambria*	2010	
KF 422	*F. proliferatum*	*O. sativa*	1973	Taiwan
KF 1329	*F. proliferatum*	*O. sativa*		Japan
KF 3584	*F. proliferatum*	*O. sativa*	2011	Thailand
KF 3416	*F. proliferatum*	*P. dactylifera*	2010	Tunisia
KF 3321	*F. temperatum*	*A. comosus*	2008	Costa Rica
KF 3488	*F. verticillioides*	*Z. mays*	2010	Poland
KF 3482	*F. verticillioides*	*Z. mays*	2010	Poland
KF 3483	*F. verticillioides*	*Z. mays*	2010	Poland
KF 3644	*F. verticillioides*	*Z. mays*	2010	Poland
KF 3537	*F. verticillioides*	*A. comosus*	2010	Costa Rica

**Figure 1:**
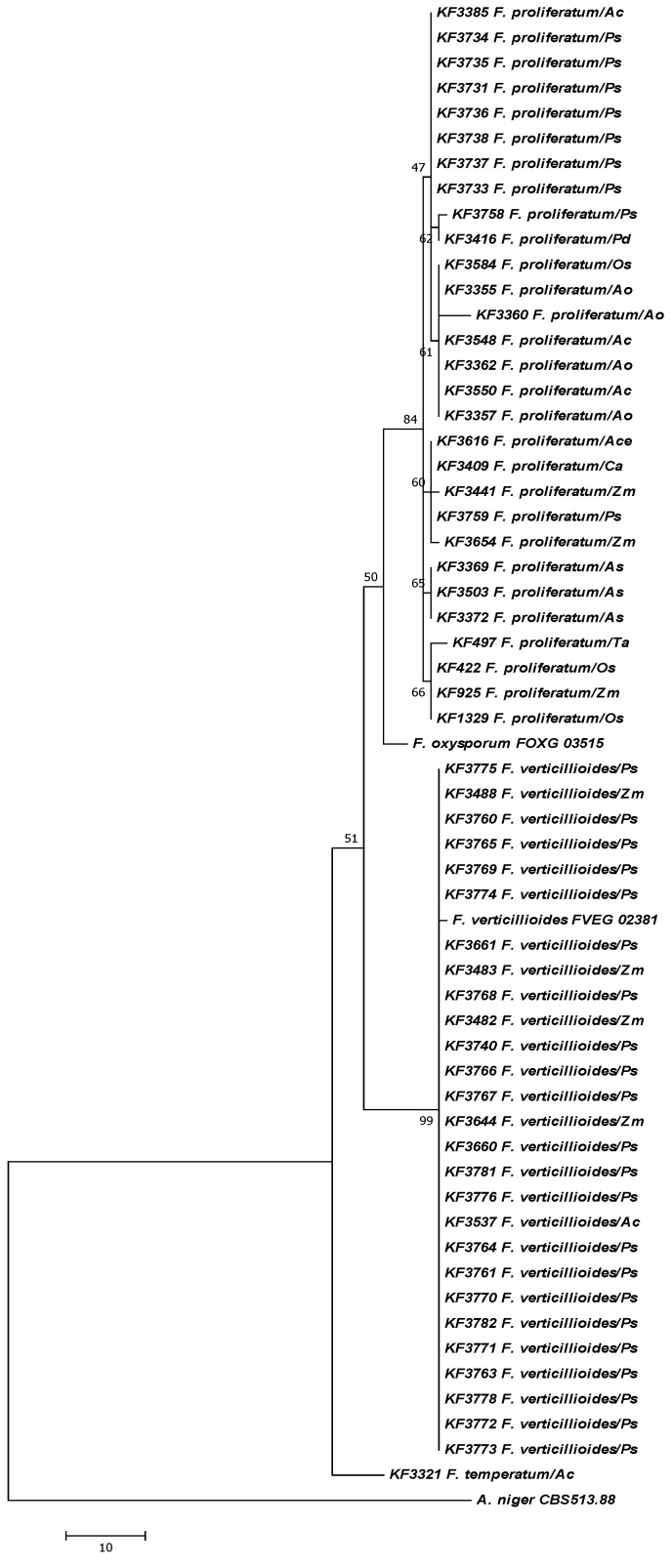
The most parsimonious tree for 57 *Fusarium* strains used in the study based on the translation elongation factor 1alpha (*tef*-1α) sequences. The reference strains of *F. oxysporum* (FOXG_03515) and *F. verticillioides* (FVEG_02381), as well as an outgroup of *A. niger* CBS 513.88 strain (GenBank Acc. NT_166526.1) were included (Fungal Genome Initiative). Maximum Parsimony approach and bootstrap test (1000 replicates) were applied. Abbreviations used for the host names: *Ac*: *Ananas comosus*; *Ace*: *Allium cepa*; *Ao*: *Asparagus officinalis*; *As*: *Allium sativum*; *Ca*: *Cambria* sp.; *Os*: *Oryza sativa*; *Pd*: *Phoenix dactylifera*; *Ps*: *Pisum sativum*; *Ta*: *Triticum aestivum*; *Zm*: *Zea mays.*

**Figure 2:**
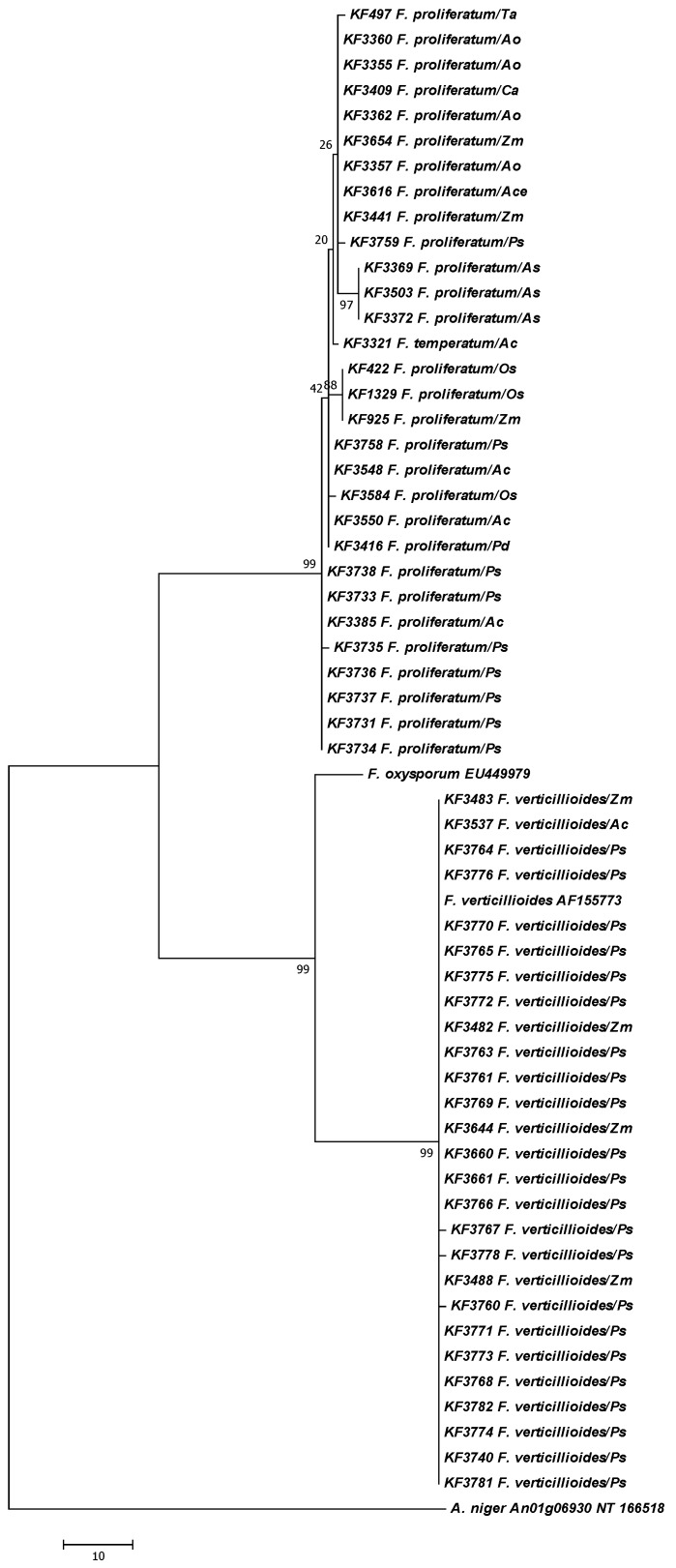
The most parsimonious tree for 57 *Fusarium* strains used in the study based on the partial sequence of the *FUM1* gene. The reference strains of *F. oxysporum* (GenBank Acc. EU449979) and *F. verticillioides* (GenBank Acc. AF155773), as well as an outgroup of *A. niger* CBS 513.88 strain (GenBank Acc. NT_166526.1) were included). Maximum Parsimony approach and bootstrap test (1000 replicates) were applied. Abbreviations used for the host names: *Ac*: *Ananas comosus*; *Ace*: *Allium cepa*; *Ao*: *Asparagus officinalis*; *As*: *Allium sativum*; *Ca*: *Cambria* sp.; *Os*: *Oryza sativa*; *Pd*: *Phoenix dactylifera*; *Ps*: *Pisum sativum*; *Ta*: *Triticum aestivum*; *Zm*: *Zea mays.*

All isolates were re-identified molecularly on the basis of the translation elongation factor 1alpha (*tef*-1α) sequence analysis and aligned to the sequences of the collection strains from different host species to evaluate the in-population genetic variability (Figure 1). Additionally, a fragment of a *FUM1* gene was partially sequenced using primers developed and validated during previous works [26,40]. Based on the multiple alignment of the sequences obtained, a dendrogram was calculated using the Maximum Parsimony approach (Figure 2). All strains under study fell firmly into the clades of *F. proliferatum* and *F. verticillioides*, discriminated on the basis of the collection strains sequences (Table 1). Moreover, a certain level of sub-specific polymorphism has been observed among the strains of *F. proliferatum* (Figure 1, Figure 2).

Having uncovered that pea seed samples quite frequently carried the dangerous *Fusarium* pathogens, a survey for fumonisin quantification has been performed for the respective seed samples of two cultivars EUREKA and TURNIA (four replicates from two localities each). Cultivars were chosen on the basis of fungal species present (Table 1). All of the samples tested contained low amounts of FBs (Table 2). However, no significant differences have been observed in FB levels between seeds containing *F. proliferatum* and *F. verticillioides* as the prevailing pathogens. A maximum amount of FBs detected in TURNIA IV from Radzików was 1.72 μg g^−1^, and the lowest (for TURNIA I from Radzików) was 0.63 μg g^−1^. FB_1_ was dominating markedly, representing more than 90% of the total amount at all times (Table 2).

**Table 2:** Fumonisin concentration (in μg g^−1^) and standard deviations (SD) in seeds of two pea cultivars (EUREKA and TURNIA) grown in 2011 season in two distinct localities of Poland (R: Radzików, W: Wiatrowo) and naturally infected with fumonisin-producing *F. verticillioides* and *F. proliferatum*.

Sample	FB_1_	FB_2_	FB_3_
EUREKA_I (W)	1.12	0.05	0.01
EUREKA _II (W)	1.34	0.07	0.02
EUREKA _III (W)	0.79	0.05	0.01
EUREKA _IV (W)	0.81	0.04	0.01
Mean ± SD	1.02 ± 0.26	0.05 ± 0.01	0.01 ± 0.01
EUREKA_I (R)	1.11	0.12	0.05
EUREKA_II (R)	0.72	0,03	0.00
EUREKA III (R)	0.45	0.12	0.08
EUREKA_IV (R)	0.63	0.04	0.01
Mean ± SD	0.73 ± 0.28	0.08 ± 0.05	0.04 ± 0.04
TURNIA_I (W)	0.85	0.11	0.04
TURNIA _II (W)	0.81	0.09	0.05
TURNIA _III (W)	0.92	0.07	0.01
TURNIA _IV (W)	0.91	0.08	0.02
Mean ± SD	0.87 ± 0.05	0.09 ± 0.02	0.03 ± 0.02
TURNIA_I (R)	0.55	0.06	0.02
TURNIA_II (R)	0.61	0.07	0.01
TURNIA_III (R)	1.27	0.14	0.08
TURNIA_IV (R)	1.48	0.15	0.09
Mean ± SD	0.98 ± 0.47	0.11 ± 0.05	0.05 ± 0.04

In order to evaluate the efficacy of the fumonisin B_1_–B_3_ biosynthesis by *F. proliferatum* and *F. verticillioides* isolates, the *in vitro* cultures on sterile rice grain were prepared [40]. Eighteen genotypes originating from TURNIA, EUREKA and SOKOLIK cultivars were analyzed. The amounts of FBs were quantified using a standardized high-performance liquid chromatography (HPLC) method (Section 4.5). Additionally, several collection strains of *F. proliferatum* and *F. verticillioides* originating from various host species [26] have been included to show the intraspecific variability of this trait (Table 3). 

**Table 3:** Means and standard deviations (SD) of fumonisins concentrations (in μg g^−1^) produced in rice cultures by *F. verticillioides* and *F. proliferatum* strains purified from infected seeds of various pea cultivars from two localities (R: Radzików, W: Wiatrowo), as well as by several collection strains of both species (please refer to [26] for more *F. proliferatum* strains).

Strain	Species	Host/cultivar/locality	FB_1_	FB_2_	FB_3_
KF 3779	*F. verticillioides*	*P. sativum*/TURNIA/W	155.92 ± 12.31	0.60 ± 0.02	5.44 ± 1.13
KF 3780	*F. verticillioides*	*P. sativum*/TURNIA/W	183.55 ± 14.03	0.98 ± 0.03	16.75 ± 2.52
KF 3760	*F. verticillioides*	*P. sativum*/TURNIA/W	317.24 ± 16.32	34.00 ± 3.15	7.01 ± 1.14
KF 3781	*F. verticillioides*	*P. sativum*/TURNIA/W	80.39 ± 7.44	0.49 ± 0.15	2.66 ± 0.09
KF 3761	*F. verticillioides*	*P. sativum*/TURNIA/W	202.29 ± 10.18	95.61 ± 8.53	37.44 ± 4.79
KF 3782	*F. verticillioides*	*P. sativum*/TURNIA/W	111.65 ± 11.59	52.35 ± 4.17	27.62 ± 5.56
KF 3731	*F. proliferatum*	*P. sativum*/TURNIA/R	111.07 ± 9.47	49.61 ± 5.22	43.58 ± 6.85
KF 3732	*F. proliferatum*	*P. sativum*/TURNIA/R	958.51 ± 21.06	271.22 ± 10.84	104.30 ± 9.48
KF 3733	*F. proliferatum*	*P. sativum*/TURNIA/R	121.65 ± 10.11	51.99 ± 6.63	37.71 ± 5.54
KF 3734	*F. proliferatum*	*P. sativum*/TURNIA/R	49.36 ± 5.33	29.26 ± 4.12	30.85 ± 3.52
KF 3735	*F. proliferatum*	*P. sativum*/TURNIA/R	845.56 ± 42.67	227.99 ± 10.47	112.65 ± 9.65
KF 3736	*F. proliferatum*	*P. sativum*/TURNIA/R	476.53 ± 25.13	227.78 ± 11.58	111.39 ± 8.83
KF 3737	*F. proliferatum*	*P. sativum*/TURNIA/R	106.09 ± 9.88	49.72 ± 5.39	43.84 ± 5.28
KF 3738	*F. proliferatum*	*P. sativum*/TURNIA/R	648.30 ± 21.56	180.68 ± 15.41	101.13 ± 4.69
KF 3660	*F. verticillioides*	*P. sativum*/EUREKA/R	78.74 ± 7.40	24.20 ± 3.30	4.68 ± 0.08
KF 3740	*F. verticillioides*	*P. sativum*/EUREKA/R	216.50 ±15.84	89.95 ± 7.17	43.33 ± 6.16
KF 3661	*F. verticillioides*	*P. sativum*/EUREKA/R	1861.52 ± 54.23	108.16 ± 7.49	40.61 ± 5.58
KF 3758	*F. proliferatum*	*P. sativum*/EZOP/W	212.02 ± 12.36	63.50 ± 8.82	44.01 ± 6.85
KF 3416	*F. proliferatum*	*P. dactylifera*	46.34 ± 5.41	26.11 ± 5.41	6.53 ± 0.08
KF 3357	*F. proliferatum*	*A. officinalis*	1536.00 ± 52.33	657.09 ± 80.35	123.72 ± 8.71
KF 3654	*F. proliferatum*	*Z. mays*	1578.04 ± 41.25	529.96 ± 69.15	91.34 ± 9.13
KF 3584	*F. proliferatum*	*O. sativa*	201.20 ± 7.69	53.60 ± 7.74	33.00 ± 4.28
KF 3409	*F. proliferatum*	*Cambria* sp.	668.72 ± 18.47	170.07 ± 25.13	70.19 ± 6.71
KF 3503	*F. proliferatum*	*A. sativum*	1186.87 ± 87.42	185.54 ± 18.53	66.24 ± 5.93
KF 3537	*F. verticillioides*	*A. comosus*	59.65 ± 6.06	19.37 ± 2.47	5.86 ± 0.98
KF 3644	*F. verticillioides*	*Z. mays*	6.12 ± 1.52	0.37 ± 0.05	0.29 ± 0.03
KF 3488	*F. verticillioides*	*Z. mays*	39.77 ± 4.15	0.96 ± 0.09	0.06 ± 0.01
KF 3482	*F. verticillioides*	*Z. mays*	273.38 ± 14.39	60.35 ± 1.11	0.88 ± 0.04
KF 3483	*F. verticillioides*	*Z. mays*	14.17 ± 2.08	0.04 ± 0.01	0.00 ± 0.00

## 3. Discussion

Seeds of only six out of twelve pea cultivars screened appeared to contain fumonisin-producing *Fusarium* species and in the seeds of EZOP and TURNIA the pathogens occurred frequently. *F. proliferatum* was predominantly occurring on cv. TURNIA and *F. verticillioides* on cv. EZOP (Table 1). Other *Fusarium* species were isolated only occasionally. Species identification was performed on the basis of the translation elongation factor 1alpha (*tef*-1α) sequence analysis. This gene is widely used in phylogenetic studies of fungi, successfully resolving most of the closely related *Fusarium* species [17,44,45]. However, in populations of some less polymorphic species, where the genotypes studied display a low level of genetic diversity, different genomic regions should be used to increase the polymorphism revealed [38,46]. 

Polyketide synthase is the essential enzyme of the fumonisin biosynthetic pathway is encoded by *FUM1* gene [47]. Using primers developed and validated during the previous works [26,40], *FUM1* gene fragments were sequenced and comparatively analyzed using all the strains included in the study. Finally, a dendrogram was calculated using the Maximum Parsimony approach to show the divergences among the strains originating from different host species. All strains formed two separate and well-supported clades of *F. proliferatum* and *F. verticillioides* (Figure 2). Furthermore, a certain level of sub-specific polymorphism has been observed among the strains in the case of *F. proliferatum* genotypes (Figure 2). In the case of *FUM* genes this observation was already reported [26,40]; however, the analysis of the pea-derived strains is presented here for the first time. It seems that the pea-derived strains of *F. proliferatum* are highly uniform and show the highest similarity level to some genotypes originating from pineapple and date palm (Figure 2). On the contrary, *F. verticillioides* strains have shown virtually no difference among the populations from different hosts. It could implicate that *F. proliferatum* displays a higher evolutionary potential. In fact, this hypothesis seems to be fairly supported by the results of analyses performed during this and the previous studies [8,26,36,40]. 

Furthermore, the sequences of the biosynthetic genes from other mycotoxin pathways have been utilized in phylogenetic studies of *Fusarium* species [38,41,42], showing considerably higher polymorphism than the commonly used conserved genes from the primary metabolic pathways. Thus, markers for secondary metabolite biosynthetic genes can be sensitive tools for the prediction of the mycotoxin presence in plant samples. Here, pea seeds of the cultivars containing *F. verticillioides* have been analyzed. All samples tested contained low amounts of FBs (Table 1), though the levels of FBs were similar in the samples of seeds containing *F. proliferatum* and *F. verticillioides*, as the prevailing pathogens. FB_1_ dominated markedly, representing more than 90% of the total amount at all times (Table 2). Moreover, there was no correlation between the frequency of the pathogen detection in particular pea cultivar samples coming from different locations and the observed fumonisin content. The efficacies of the fumonisin B_1_–B_3_ biosynthesis by eighteen genotypes of *F. proliferatum* and *F. verticillioides* strains originating from TURNIA, EUREKA and SOKOLIK cultivars, were evaluated on the basis of the *in vitro* cultures on sterile rice grain [40]. Concentrations of fumonisins produced on this substrate were lower than these observed on maize kernels [16,36], but still exceeded 1.5 mg g^−1^ for some strains (Table 3). This difference may be related to the starch content of maize grain. Another possible reason is the crucial role of fumonisins during maize infestation, which has not been proven for other host-pathogen systems yet [48]. Generally, in rice cultures *F. proliferatum* genotypes produced higher amounts of FBs than *F. verticillioides* strains, however, the most efficient strain was *F. verticillioides* strain KF3661 from cultivar EUREKA (Table 3). Remarkably, the ratios between FB_1_, FB_2_ and FB_3_ have shown higher variance than in the case of pea seed analyses. Some *F. proliferatum* genotypes accumulated FB_2_ in amounts measuring as much as 1/3 of FB_1_ level (e.g., KF 3357 and KF 3654). Conversely, few *F. verticillioides* genotypes (KF 3780 and KF 3781) synthesized virtually no FB_2_ with simultaneous higher amounts of FB_3_. Similar results were obtained for *F. verticillioides* strains originating from maize, though, FB_2_ and FB_3_ were almost absent there (e.g., KF 3644, KF 3488). In fact, for those incidences also FB_1_ was produced in very low amounts. Moreover, for several medium-producing strains (e.g., *F. proliferatum* KF 3731 and KF 3737) the levels of FB_2_ and FB_3_ were similar and reached almost a half of the FB_1_ amounts (Table 3).

## 4. Experimental Section

### 4.1. Seed Samples and Purification of Fungal Strains

Twelve pea cultivars (EUREKA, EZOP, GWAREK, HUBAL, LASSO, MEDAL, SANTANA, SOKOLIK, TARCHALSKA, TURNIA, WENUS and WIATO) were grown in two localities in Central Poland (Radzików and Wiatrowo) in 2011 season. Each genotype was sown in four randomly localized replicates, which were subsequently considered as a single sample. Ten cultivars originating from Poland, one from Germany (SANTANA) and one from Belgium (LASSO), registered between 1998 and 2011, were tested for the fungi occurrence. Fifty seeds were surface-sterilized with 0.5% sodium hypochlorite for 30 s, rinsed with sterile water and plated on a water-soaked paper in the sterile Petri-dishes for seven days. After that time seeds infected with filamentous fungi were transferred onto new plates with potato dextrose agar (PDA) medium. Hyphae tips were passaged several times on clean PDA plates to purify the strains, which were then inoculated on the synthetic nutrient agar (SNA) medium for microscopic species identification and also on the PDA plates to collect the mycelia for the extraction of the genomic DNAs. 

### 4.2. *Fusarium* Species Identification

Only *Fusarium-*infected seed samples were considered in further analyses. *Fusarium* species were identified morphologically according to Nelson *et al.* [49] manual. Optical microscope (Olympus, Tokyo, Japan) and 100× of total magnification was used for observation of the presence of microconidia and the nature of the conidiogenous cells.

### 4.3. Molecular Analyses: DNA Extraction, Primers and PCR Conditions

Genomic DNA extraction was done using a Cetyltrimethyl Ammonium Bromide (CTAB-based method [50]. Partial sequence of the *tef-*1α gene was amplified using Ef728M (CATCGAGAAGTTCGAGAAGG)/Tef1R (GCCATCCTTGGAGATACCAGC) primer combination [40]. Fum1F1 (CACATCTGTGGGCGATCC)/Fum1R2 (ATATGGCCCCAGCTGCATA) primers were used for the amplification of *FUM1* gene fragments [26,40]. The polymerase chain reaction (PCR) was done in 20 μL aliquots using PTC-200 and C-1000 thermal cyclers (BioRad, Hercules, CA, USA). Each sample contained 1 unit of Phire II HotStart Taq DNA polymerase (Finnzymes, Espoo, Finland), 4 μL of 10× PCR buffer, 12.5 pmol of forward/reverse primers, 2.5 mM of each dNTP and about 20–50 ng of fungal DNA. PCR conditions were as follows: 30 s at 98 °C, 35 cycles of (5 s at 98 °C, 5 s at 63 °C, 15 s at 72 °C) and 1 min at 72 °C. Amplicons were electrophoresed in 1.5% agarose gels (Invitrogen, Carlsbad, CA, USA) with ethidium bromide.

### 4.4. DNA Sequencing, Analysis and Phylogeny Reconstruction

PCR-amplified DNA fragments were purified for sequence analysis with exonuclease I (Epicentre, Madison, WI, USA) and shrimp alkaline phosphatase (Promega, Madison, WI, USA) using the following program: 30 min at 37 °C, followed by 15 min at 80 °C. Both strands were labeled using the BigDyeTerminator 3.1 kit (Applied Biosystems, Foster City, CA, USA), according to Błaszczyk *et al.* [51] and the manufacturer’s instructions. Labeled fragments were precipitated with ethanol to remove the remains of the reagents. Sequence reading was performed using Applied Biosystems equipment.

Sequences were compared to the NCBI GenBank-deposited sequences to confirm the correct morphological species identification using BLASTn algorithm (MEGABLAST). The collection strains of *F. proliferatum* and *F. verticillioides* originating from different host species were included for comparative analysis (Table 3).

The sequences of the PCR products were aligned with ClustalW algorithm. Phylogenetic relationships were reconstructed with MEGA4 software package [52] using Maximum Parsimony approach (Closest Neighbor Interchange heuristics). No gap-containing positions were considered in phylogeny analysis. All reconstructions were tested by bootstrapping with 1000 replicates. 

### 4.5. Fumonisin Quantification

Ten dried pea seeds of each sample (about 5.5 g in weight) were ground using a steel ball mill (Tissue Lyser II). Homogenized plant material was then subjected to the fumonisin extraction procedure (see below).

For toxin quantification rice cultures were prepared for individual *Fusarium* isolates [42]. Long-grain white rice samples were used (50 g per flask with the addition of 12.5 mL of sterile water), left overnight and sterilized by autoclaving the next day. The rice samples were subsequently inoculated with 4 cm^2^ of 7-day-old mycelium on potato dextrose agar (PDA) medium. Culture humidity was kept around 30% for 14 days. Then the cultures were dried in room temperature.

Standards of pure FB_1_, FB_2_ and FB_3_; (Sigma, St. Louis, MO, USA). Acetonitrile, methanol (HPLC grade), disodium tetraborate, 2-mercaptoethanol were purchased from Sigma-Aldrich. Potassium hydroxide, acetic acid, *o*-phosphoric acid were purchased from POCh (Gliwice, Poland). Water for the HPLC mobile phase was purified using a Milli-Q system (Millipore, Bedford, MA, USA).

Samples (5 g) of plant material were homogenized for 3 min in 10 mL of methanol-water (3:1, *v*/*v*) and filtered through Whatman No. 4 filter paper. The extract was adjusted to pH 5.8–6.3 using 0.1 mol L^−1^ KOH. A SAX cartridge was attached to the solid-phase extraction (SPE) manifold unit (Supelco, Bellefonte, PA, USA), following the method described by Waśkiewicz *et al.* [30]. The *o*-phosphoric acid (OPA) reagent (20 mg per 0.5 mL of methanol) was prepared and diluted with 2.5 mL of 0.1 mol L^−1^ disodium tetraborate (Na_2_B_4_O_7_ × 10 H_2_O). It was then combined with 25 μL 2-mercaptoethanol, which was added to the solution. The FBs standards (5 μL) or extracts (20 μL) were derivatized with 20 μL or 80 μL of the OPA reagent. The reaction mixture (10 μL) was injected onto an HPLC column 3 min later. After filtration through a 0.45 μm Waters HV membrane, methanol-sodium dihydrogen phosphate (0.1 mol L^−1^ in water) solution (77:23, *v*/*v*), adjusted to pH 3.35 with *o*-phosphoric acid, was used as a mobile phase with a flow rate of 0.6 mL min^−1^. 

A Waters 2695 HPLC instrument (Waters Division of Millipore, Milford, MA, USA) with an X-Bridge column (3.9 mm × 100 mm) and a Waters 2475 fluorescence detector (λ_EX_ = 335 nm, λ_EM_ = 440 nm) were used for determining the quantity of metabolites. The detection limit was 10 ng g^−1^ for FBs. Positive results (on the basis of retention time) were confirmed by HPLC analysis of standards and compared with the relevant calibration curves (correlation coefficients for FB_1_, FB_2_ and FB_3_ were 0.9987, 0.9991 and 0.9979, respectively). Recoveries for fumonisins were 94%, 98% and 89%, respectively, which were measured in triplicate by extracting the mycotoxins from blank samples spiked with 10–100 ng g^−1^ of the compound. The relative standard deviations (RSD) were below 7%.

## 5. Conclusions

It can be concluded that the pea-originating *F. proliferatum* and *F. verticillioides* isolates produced less fumonisins than the genotypes originating from different host species, like maize, garlic or asparagus [8,26,38]. Also, some pineapple-derived *F. proliferatum* strains were found to be very efficient FB-producers [33]. Comparing the genetic diversity of the two species, *F. verticillioides* appears as more uniform, but still, the strains differed remarkably in FBs synthesis. Taking into account the divergence of the *FUM1* gene in relation to the variance observed in the amounts of FBs produced *in vitro*, it is the differential regulation pattern governing this variance, rather than the structural divergences of the essential fumonisin biosynthetic genes. This hypothesis, however, needs to be confirmed by conducting additional experiments, e.g., by analyzing the transcription levels of the essential *FUM* genes.
